# Transcription Dynamics and DNA Methylation Responses to Growth Modification

**DOI:** 10.1007/s10126-025-10476-3

**Published:** 2025-06-12

**Authors:** Kris A. Christensen, Étienne Collette, Danielle Perley, Dionne Sakhrani, Annette F. Muttray, Rosalind A. Leggatt, Carlo A. Biagi, Ben F. Koop, Robert H. Devlin

**Affiliations:** 1 Fisheries and Oceans Canada, West Vancouver, BC Canada; 2 University of Victoria, Victoria, BC Canada; 3 Canadian Centre for Computational Genomics (C3G), Montréal, QC Canada

**Keywords:** Feed-deprivation, Growth hormone, Transgenic, Gene regulation, Promoter, Epigenetic

## Abstract

To better understand how gene transcription is controlled under different physiological and environmental conditions, we assessed transcription and methylation responses in a vertebrate system (salmon) where growth can be manipulated in response to growth hormone transgenesis or under satiated, feed-deprived, and re-fed experimental conditions. In both transgenic and non-transgenic liver tissue, methylation of gene promoters was negatively associated with transcription at all measured times. However, the changes in promoter methylation among time-points did not generally correlate with changes in gene transcription among treatments. The results from this study suggest that only a subset of genes are readily responsive to changes in promoter methylation for a given environmental shift such as feed-deprivation or a physiological shift such as growth hormone transgenesis (i.e., genotype-by-environment interactions). The present study also reveals the complexity of how one tissue responds to alterations in internal changes in physiology (growth modification by growth hormone transgenesis; genotype), external environmental conditions (food availability; environment), and their interactions (genotype-by-environment effects).

**Supplementary Information:**

The online version contains supplementary material available at 10.1007/s10126-025-10476-3.

## Introduction

Gene transcription is an emergent property of a complex interplay of multiple factors. This interplay can involve epigenetic regulation (Gibney and Nolan 2010), regulatory miRNAs (Pu et al. 2019), proteins (e.g., transcription factors and histones), DNA sequences such as enhancer regions (Pennacchio et al. 2013), and enzymes (e.g., RNA polymerase II). As the entire diversity of cell types, organs, and even organisms are dependent on the establishment of unique gene expression patterns within a cell, it is not surprising that gene transcription has so many layers of regulation and necessary components. Researchers have been peeling back each of these layers to uncover how gene transcription is regulated for at least five decades (Cramer 2019) with the hope to eventually understand how cell identity and function begin to emerge from the interplay of all these components.

As examples of the underlying complexity of gene transcription, consider transcription factors and enhancer and silencer regions. In humans, there are around 1,600 transcription factors that influence gene transcription in a very dynamic manner – with a single transcription factor able to regulate different genes depending on cell type (Lambert et al. 2018). The number of transcription factors is relatively small, however effects can be significant due to their interactions and further when compared to the estimated hundreds of thousands of enhancer and silencer sequences scattered throughout the human genome (Pennacchio et al. 2013).

Gene duplication likely played a role in increasing gene regulatory diversity and the evolution of these mechanisms. A whole-genome duplication in the ray-finned fishes (Amores et al. 1998), and another in salmonids (Allendorf and Thorgaard 1984), increased the number of transcription factors and enhancer sequences in coho salmon (*Oncorhynchus kisutch*), a species with around 2,035 transcription factors (Mulugeta et al. 2019). The possible interactions between transcription factors and enhancer sequences alone sets a minimum scale for how elaborate the regulation of gene transcription must really be.

Another element of the gene transcription regulatory machinery is DNA methylation (often categorized under epigenetic regulation). DNA methylation is thought to influence protein binding to DNA – sometimes repressing gene transcription with increased methylation of CpG nucleotide pairs in the promoter region of a gene (reviewed in (Gibney and Nolan 2010) and (Greenberg and Bourc’his 2019)). Over 80% of all CpGs outside of CpG islands are commonly methylated (Gibney and Nolan 2010). Generally, the promoter regions of active genes have low levels of methylation, but conversely the rest of an active gene body might have enriched levels of methylation (Greenberg and Bourc’his 2019).

Studies of gene transcription regulation by DNA methylation in salmonids (e.g., (Burgerhout et al. 2017; Moghadam et al. 2017; Uren Webster et al. 2018; Beemelmanns et al. 2021; Christensen et al. 2021, p.; Mukiibi et al. 2022; Freij et al. 2024)) often have inconsistent associations with changes in transcription and methylation among treatments – with genome-wide analyses suggesting that only a small fraction of gene transcription changes could possibly be regulated as a result of changes in methylation. This is not surprising given the diversity and complexity of gene transcription regulation. Even when gene transcription is associated with changes in methylation, other gene regulatory elements may be influencing both changes in methylation and gene transcription.

Stable, global gene transcription in salmonids does appear to have a strong correlation with methylation (e.g., (Mukiibi et al. 2022)), as opposed to gene transcription that significantly changes among treatments. This suggests that methylation could play a larger role in long-term gene transcription regulation than in gene transcription changes that might be expected during short-term environmental changes. Categorizing which genes are responsive to immediate gene transcription regulation by methylation would help us to better understand methylation as a gene regulatory mechanism. Substantial nutritional and energy responses in salmonids to dietary conditions provides an opportunity to examine to what degree methylation plays a role in gene regulatory responses that in turn affect the physiology of the organism.

As with many other organisms, wild salmon regularly encounter variable food abundance in nature, and can be food-deprived during periods of low food abundance followed by intense re-feeding when food becomes available. This is especially true during autumn and winter when food sources become much more limited (Simpkins and Hubert 2000). Rainbow trout (*Oncorhynchus mykiss*) can survive at least 147 days without food depending on the initial size of the trout (Simpkins et al. 2004), a likely evolutionary adaptation to the extended feed deprivation encountered during winter months. Under aquaculture conditions, growth compensation following feed-deprivation has been actively researched as a means for increasing growth rate (Hornick et al. 2000; Won and Borski 2013; Abernathy et al. 2015).

Growth hormone (GH) transgenesis has been successfully applied to salmonid species, including Atlantic salmon (*Salmo salar*), coho salmon, and rainbow trout (Du et al. 1992; Devlin et al. 2001). GH-transgenic coho salmon have a much elevated metabolism and growth rate compared to non-transgenic strains when provided with sufficient food (Devlin et al. 1995). Restricting feed, and thereby decreasing the available energy for GH-transgenic coho salmon, depresses their metabolism such that their growth potential becomes similar to that of wild-type coho salmon (Overturf et al. 2010). Fully-fed transgenic and domesticated coho salmon showed significantly increased levels of expression of metabolic genes (e.g., *pyruvate dehydrogenase, aspartate aminotransferase*, and *PPARγ*) as well as the oxidative stress gene glutathione peroxidase in the liver compared to restricted ration-fed transgenics or non-transgenic coho salmon (Overturf et al. 2010). In a three-month food deprivation study with GH-transgenic and non-transgenic coho salmon (Abernathy et al. 2015) researchers observed that non-transgenic salmon had an overall greater rate and extent of protein utilization than transgenic salmon with five genes related to liver metabolism differentially regulated (gluconeogenesis, lipogenesis, lipolysis, and glycolysis: *carnitine palmitoyltransferase, fatty acid synthase, glucose-6-phosphatase, glucose-6-phosphate dehydrogenase, glucokinase*). GH-transgenic coho salmon also have increased utilization of carbohydrates for energy production and lipid synthesis (Leggatt et al. 2009).

In the current study, we utilized a growth hormone transgenic salmon model (Devlin et al. 2004) that causes a very strong transformation in growth rate relative to non-transgenic salmon, and we also varied feed availability (energy) to induce changes in gene expression with the objective of improving our understanding of the role of gene expression control under different genetic and environmental conditions. From previous research, we expected changes in gene expression and metabolic pathways due to differences between GH-transgenic and non-transgenic coho salmon and feed availability (Raven et al. 2006, 2008; Rise et al. 2006; Higgs et al. 2009; Leggatt et al. 2009; Christensen et al. 2021). However, our main objectives here were to investigate 1) if there was a correlation between gene transcription and promoter methylation (at single time-points, i.e., “static methylation”), and 2) if changes in methylation also correlated with changes in gene transcription.

## Results

### Feed Deprivation and Re-feeding

Sampled (liver tissue) transgenic and non-transgenic coho salmon were size-matched before feed-deprivation (Time 0) and did not significantly vary in weight (based on a two-way ANOVA, *p* = 0.48). This was also the case for the duration of the experiment (*p* = 0.34, Figure S1 and Table S1). A small but significant genotype effect was observed for length (20.39 cm vs. 19.77 cm, *p* = 0.038, two-way ANOVA). We note that the transgenic and non-transgenic salmon were both included to better understand how the results may vary among salmon with substantially different physiologies rather than as direct comparisons and the difference in length is unimportant for this comparison. We also note that both non-transgenic and transgenic salmon lost weight during the feed deprivation phase, whereas non-transgenic salmon showed elevated (catch-up) growth relative to transgenic salmon, which displayed steady growth (Figure S1 and Table S1).

Sample pooling of liver tissue was used to reduce sequencing cost (see Methods). However, pooled samples unintentionally differed in size (Table S1), and this could have increased the variation in gene expression and methylation. Fortunately, replicates of each pool generally clustered well together in PCAs (see below).

### RNA-seq Analysis

After sequencing, RNA-seq paired-end read counts ranged from 48,447,676 to 122,623,249 per pool of two samples before trimming. These were not significantly different among treatments or between groups (using a two-tailed Welch’s t-test). After trimming, 85–95.3% of the reads aligned to the genome assembly (Figure S2a). Most of these reads mapped to exons (88–91%). Pooled sample replicates clustered together in an MDS plot except for one of the transgenic pooled samples after refeeding (Figure S2b-c). This suggests that even though there was diversity in pooling samples of different sizes (Table S2), it did not appear to drastically influence gene expression (Figure S2b-c). This could, however, introduce variability that might mask more subtle associations of methylation and gene transcription (see below).

**Fig. 1:**
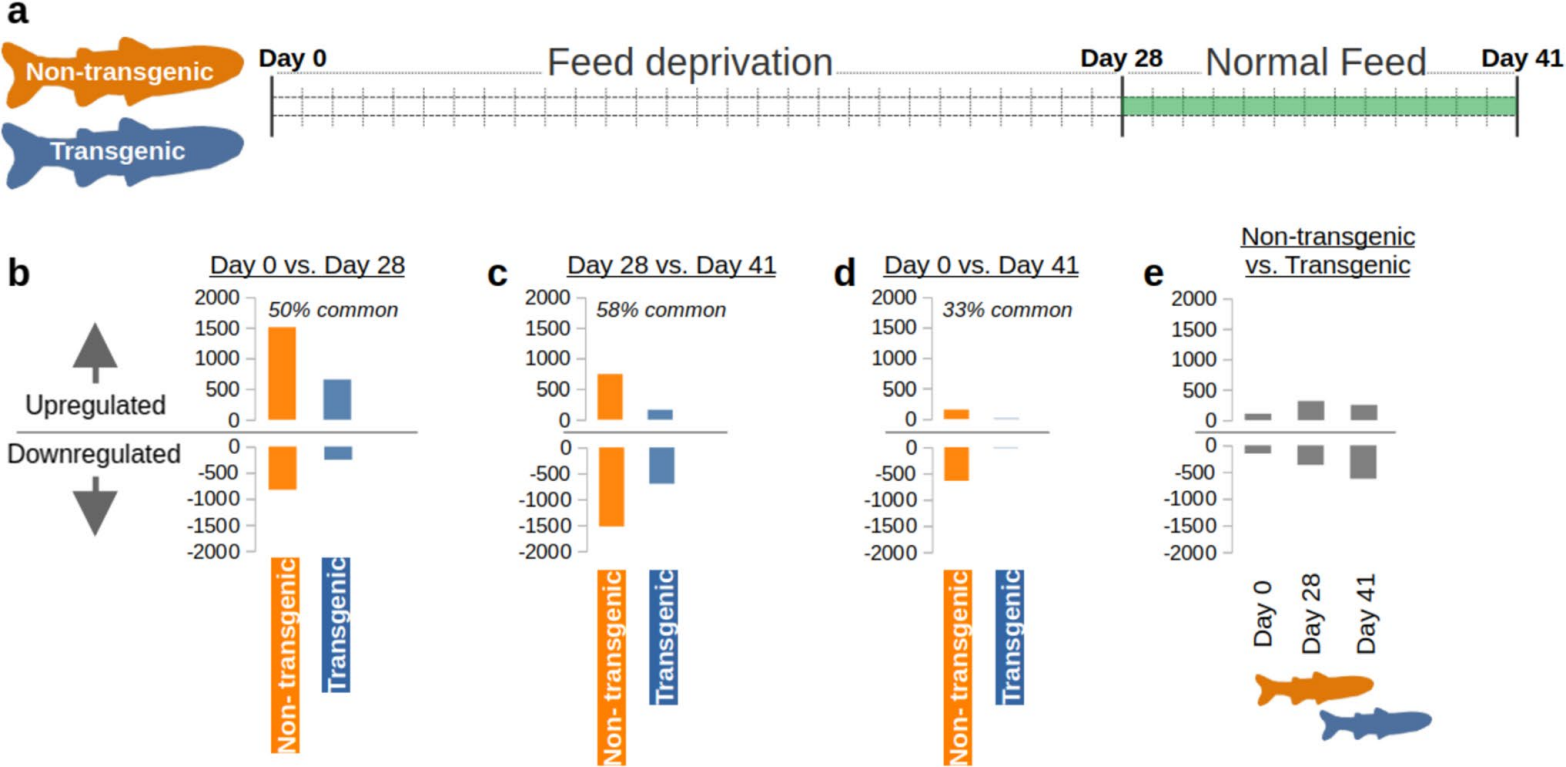
Differentially expressed gene (DEG) counts among treatments and between salmon genotypes. **a** Timeline of the study (fed—Day 0, feed-deprivation—Day 28, re-fed—Day 41). **b** Comparison between fed (Day 0) and feed-deprivation (Day 28). The number of DEGs for non-transgenic (left/orange) and transgenic salmon (right/blue) are shown in the bar plot (top—upregulated, bottom—downregulated, shown in negative values for visualization). **c** Comparison between feed-deprivation (Day 28) and re-feeding (Day 41). **d** Comparison between fed (Day 0) and re-feeding (Day 41). **e** Comparison between non-transgenic and transgenic salmon at each time point

The transcription of many genes in the liver was significantly influenced by the treatments in this study (Fig. 1b-d). This ranged from 0.08% to 8.39% of all transcripts depending on the comparison (Table 1). Fewer differentially expressed genes (DEGs) were identified among transgenic salmon than non-transgenic salmon (Fig. 1b-d). The percent of DEGs that overlapped in both non-transgenic and transgenic salmon ranged from 33–58% depending on the comparison (Fig. 1b-d). Before feed-restriction (Day 0), the combined number of DEGs between transgenic and non-transgenic salmon were at their lowest but increased during food-deprivation and further increased after re-feeding (Fig. 1e, Table 1).

**Table 1:** Counts of differentially expressed gene transcripts between treatments and groups

Comparison	Down regulated count	Up regulated count
Transgenic Day 0 vs. Day 28	253 (0.91%)	652 (2.34%)
Transgenic Day 0 vs. Day 41	11 (0.04%)	10 (0.04%)
Transgenic Day 28 vs. Day 41	700 (2.51%)	162 (0.58%)
Non-transgenic Day 0 vs. Day 28	829 (2.98%)	1508 (5.41%)
Non-transgenic Day 0 vs. Day 41	639 (2.29%)	156 (0.56%)
Non-transgenic Day 28 vs. Day 41	1524 (5.47%)	740 (2.66%)
Transgenic vs. non-transgenic Day 0	154 (0.55%)	108 (0.39%)
Transgenic vs. non-transgenic Day 28	367 (1.32%)	317 (1.14%)
Transgenic vs. non-transgenic Day 41	627 (2.25%)	253 (0.91%)

Percent of all transcripts in parentheses (27,856 transcripts assayed). Day 0 = fed, Day28 = feed-deprived, Day 41 = re-fed

GO term enrichment analyses allowed us to reveal physiological insights into liver transcription during the different treatments and how the GH transgene influenced transcription (File S1). Feed-deprivation caused an increase in the transcription of genes involved in proteolysis for both non-transgenic and transgenic salmon as well as a decrease in transcription of genes related to the cell cycle in liver tissue (File S1). After re-feeding, transcription for cell cycle genes increased for both (most returning to a baseline), but the proteolysis GO category was not significantly enriched after re-feeding, which we expected to return to baseline. A major difference between transgenic and non-transgenic salmon was how the transcription of genes related to the immune system and apoptosis fluctuated in the non-transgenic salmon, but not in the transgenic salmon (File S1). Transcription of genes related to immune system processes increased during feed-deprivation and did not return to previous levels after re-feeding for non-transgenic salmon.

### Whole Genome Bisulfite Sequencing (WGBS) Analysis

WGBS paired-end read counts ranged from 457,713,798 to 694,132,290 per pool of two samples before trimming. After trimming, 55–61.55% of the reads aligned to the genome assembly with an average coverage ranging from 12x to 20x. No significant difference was identified among treatments or groups for read alignment count (two-tailed Welch’s t-test). The bisulfite conversion ratio was over 0.99 for all samples. There were from 19,513,913 CpG loci to 29,040,542 CpG loci with 10 × coverage per pool. Replicates clustered based on CpGs, with transgenic and non-transgenic samples separated on the first dimension (Figure S3a-b). Transgenic salmon samples had considerable overlap in methylation patterns, suggesting that the variability in pooling samples with different sizes (Supplemental Table S2) could have influenced methylation in these samples. The total number of differentially methylated CpGs ranged from 1,915–35,268 among treatments and between non-transgenic and transgenic salmon (Table 2). As with gene transcription, there were more differentially methylated CpGs in non-transgenic salmon.

**Table 2:** Differentially methylated CpGs in the genome

Comparison	Total	Hypomethylated	Hypermethylated
Transgenic Day 0 vs. Day 28	3410	1860	1550
Transgenic Day 0 vs. Day 41	1915	819	1096
Transgenic Day 28 vs. Day 41	8778	3791	4987
Non-transgenic Day 0 vs. Day 28	14,132	5718	8414
Non-transgenic Day 0 vs. Day 41	13,011	6442	6569
Non-transgenic Day 28 vs. Day 41	2964	1807	1157
Transgenic vs. non-transgenic Day 0	17,800	8418	9382
Transgenic vs. non-transgenic Day 28	35,268	20,421	14,847
Transgenic vs. non-transgenic Day 41	17,755	9657	8098

Day 0 = fed, Day28 = feed-deprived, Day 41 = re-fed

**Fig. 2:**
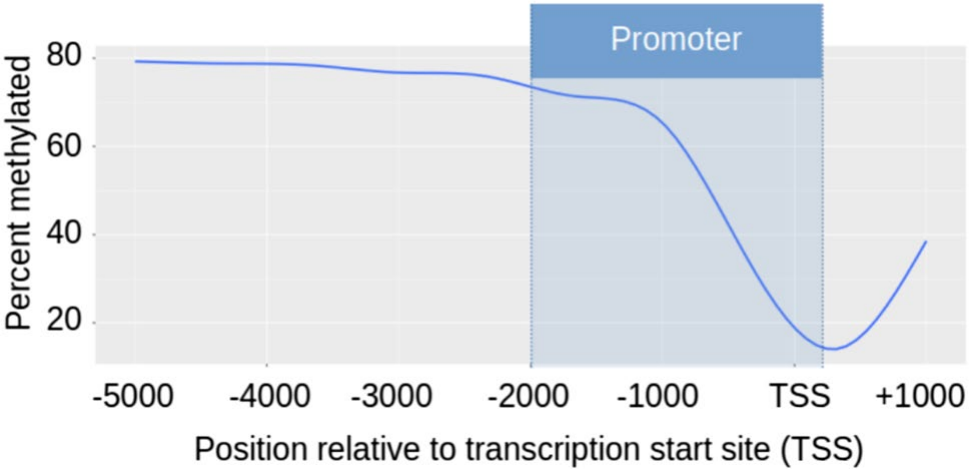
Promoter methylation. The relationship between the transcription start site (TSS) and methylation for all genes in the liver tissue of fed, non-transgenic salmon. All CpG sites within 5000 bp before the TSS and 1000 bp after were used in this analysis. The promoter region chosen for this study is highlighted. The *X*-axis represents the distance of each CpG from the transcription start site of the closest respective gene (individual CpGs were not plotted as they were too numerous). The *Y*-axis, represents the percent of methylated CpGs relative to the total CpGs. The ggplot2 (Wickham 2016) command geom_smooth was used to model the data for this plot (“gam” model)

In promoter regions, methylation fluctuated based on the position of CpG loci relative to transcription start sites, TSS (Fig. 2, Figure S4). At positions near the TSS, methylation decreased, reaching the lowest methylation ~ 200 bp downstream of a gene (~ 15% methylation). Further away, methylation increased until ~ 80% (Fig. 2).

**Fig. 3:**
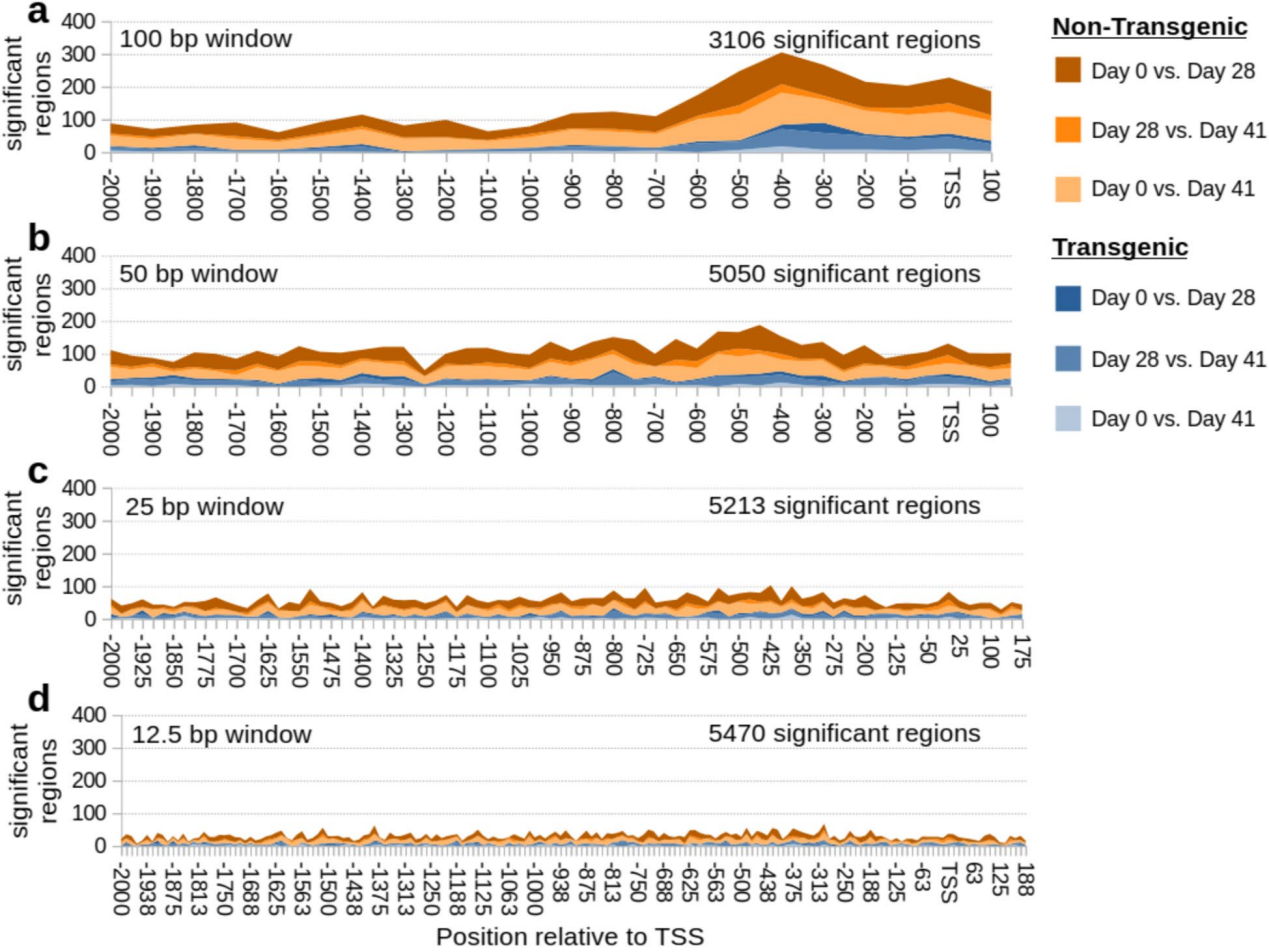
Differentially methylated regions (DMR) in the promoter. Stacked area plot of DMRs in 100 bp windows (**a**), 50 bp windows (**b**), 25 bp windows (**c**), and 12.5 bp windows (**d**)

With a window size of the whole promoter (2000 bp before and 200 bp after the TSS), only 11–56 gene promoters were significantly differentially methylated (Table 3). This increased after reducing the window size in the promoter region (Fig. 3a-d), with 3106–5470 significantly differentially methylated regions (DMRs) based on the window size. Note that multiple DMRs from the same gene are possible. With a 100 bp window size, there was a clear enrichment of differentially methylated regions from −600 bp to + 200 bp of the TSS.

**Table 3:** Counts of differentially methylated promoters

Comparison	Promoter hypo, hyper-methylation
Transgenic Day 0 vs. Day 28	5, 6
Transgenic Day 0 vs. Day 41	5, 9
Transgenic Day 28 vs. Day 41	8, 13
Non-transgenic Day 0 vs. Day 28	7, 18
Non-transgenic Day 0 vs. Day 41	15, 17
Non-transgenic Day 28 vs. Day 41	12, 16
Transgenic vs. non-transgenic Day 0	15, 15
Transgenic vs. non-transgenic Day 28	35, 21
Transgenic vs. non-transgenic Day 41	26, 19

Day 0 = fed, Day28 = feed-deprived, Day 41 = re-fed

### Overlap of DEGs and DMRs

**Fig. 4:**
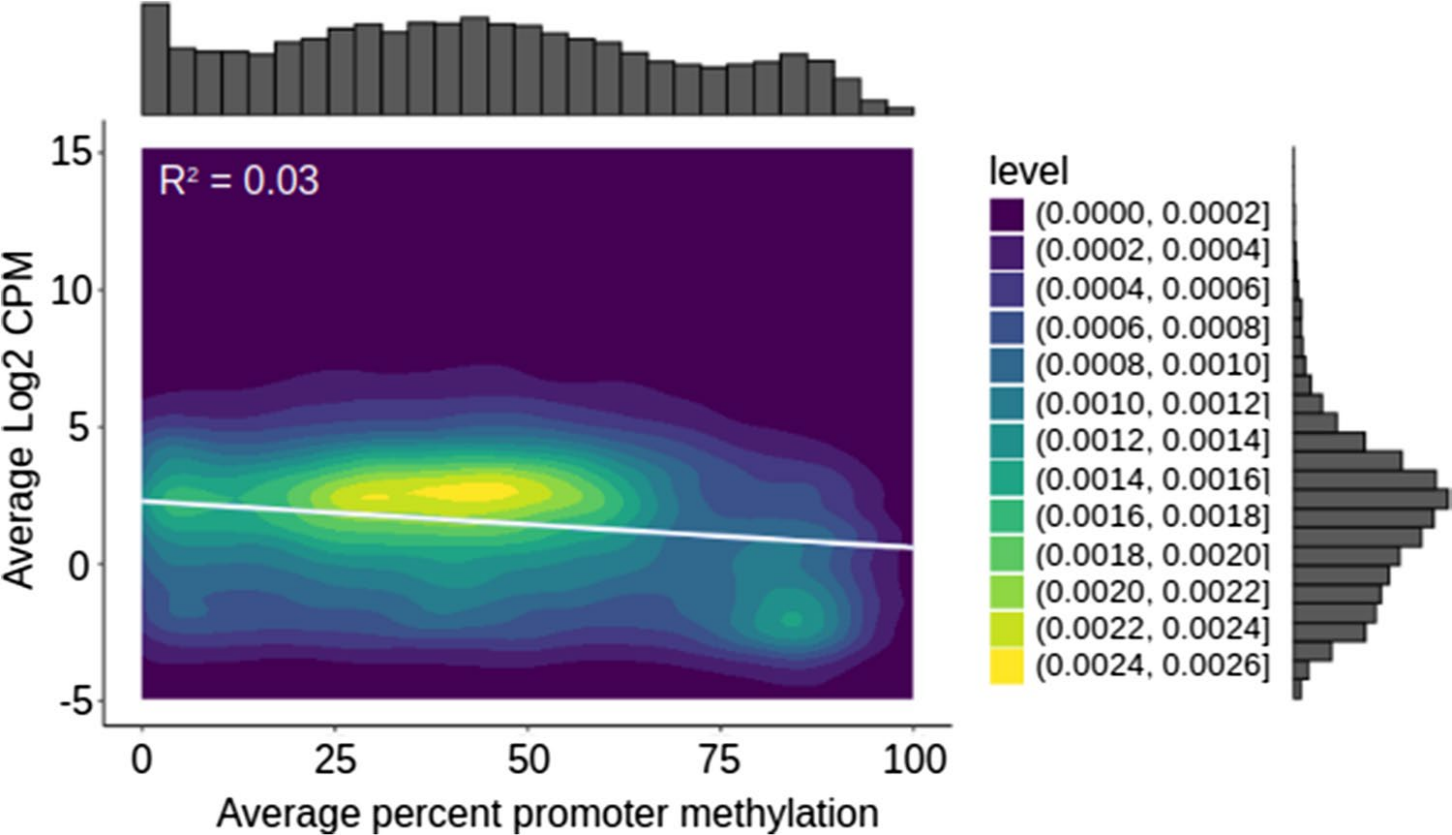
Association of promoter methylation and gene transcription. The relationship between the promoter region methylation (2000 bp upstream of the transcription start site and 200 bp downstream) and gene transcription (log2 of the counts per million mapped reads—log2 CPM) from the liver tissues of fed non-transgenic salmon (see Figure S5 for others). Only genes with values for both were plotted. The ggplot2 command geom_density2 d_filled and the ggExtra package (Attali and Baker 2022) were used to generate the plot (with linear regression line from ggpmisc package-white). Lighter (yellow) regions represent a higher density of gene promoters and their corresponding gene transcription

**Fig. 5:**
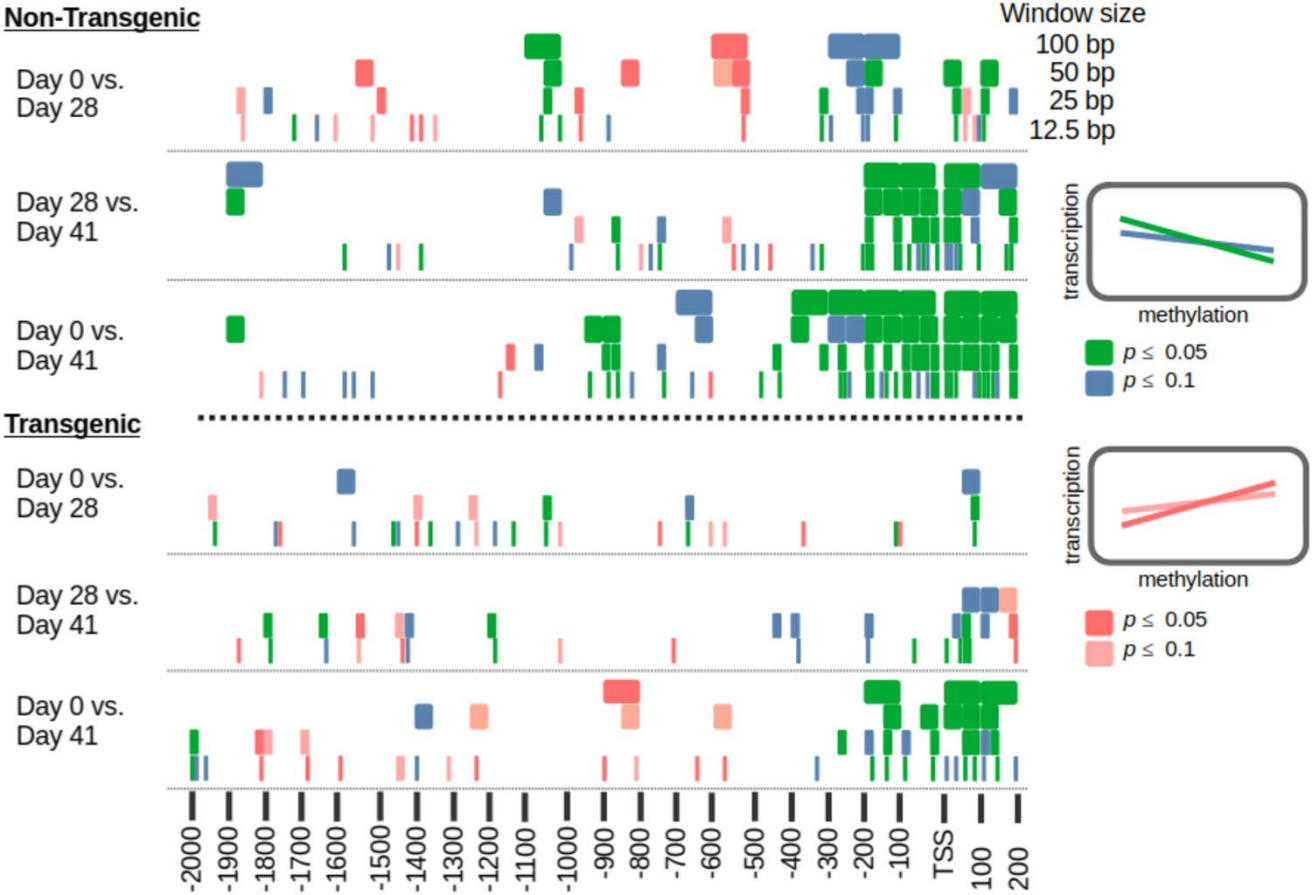
Significant associations of promoter methylation and gene transcription in different window sizes. For each comparison within a salmon genotype (noted on the *Y*-axis), significant associations of promoter methylation and gene transcription were marked based on the windows position in the promoter (*X*-axis). A key to determine the direction and significance level of an association is provided on the right

In the promoter region (defined here between 2000 bp before the TSS and 200 bp after), an inverse relationship was observed between gene transcription and methylation (Fig. 4, Figure S5), with lower gene transcription at higher levels of methylation. In smaller windows of the promoter, most significant associations were from −400 bp to + 200 bp of the TSS (Fig. 5). Transgenic salmon had fewer windows with significant associations, and in both salmon genotypes, there were more associations in the comparison between Day 0 and Day 41 than the other comparisons. When there were significant associations, the majority of genes were not involved (e.g., Figure S6).

**Fig. 6:**
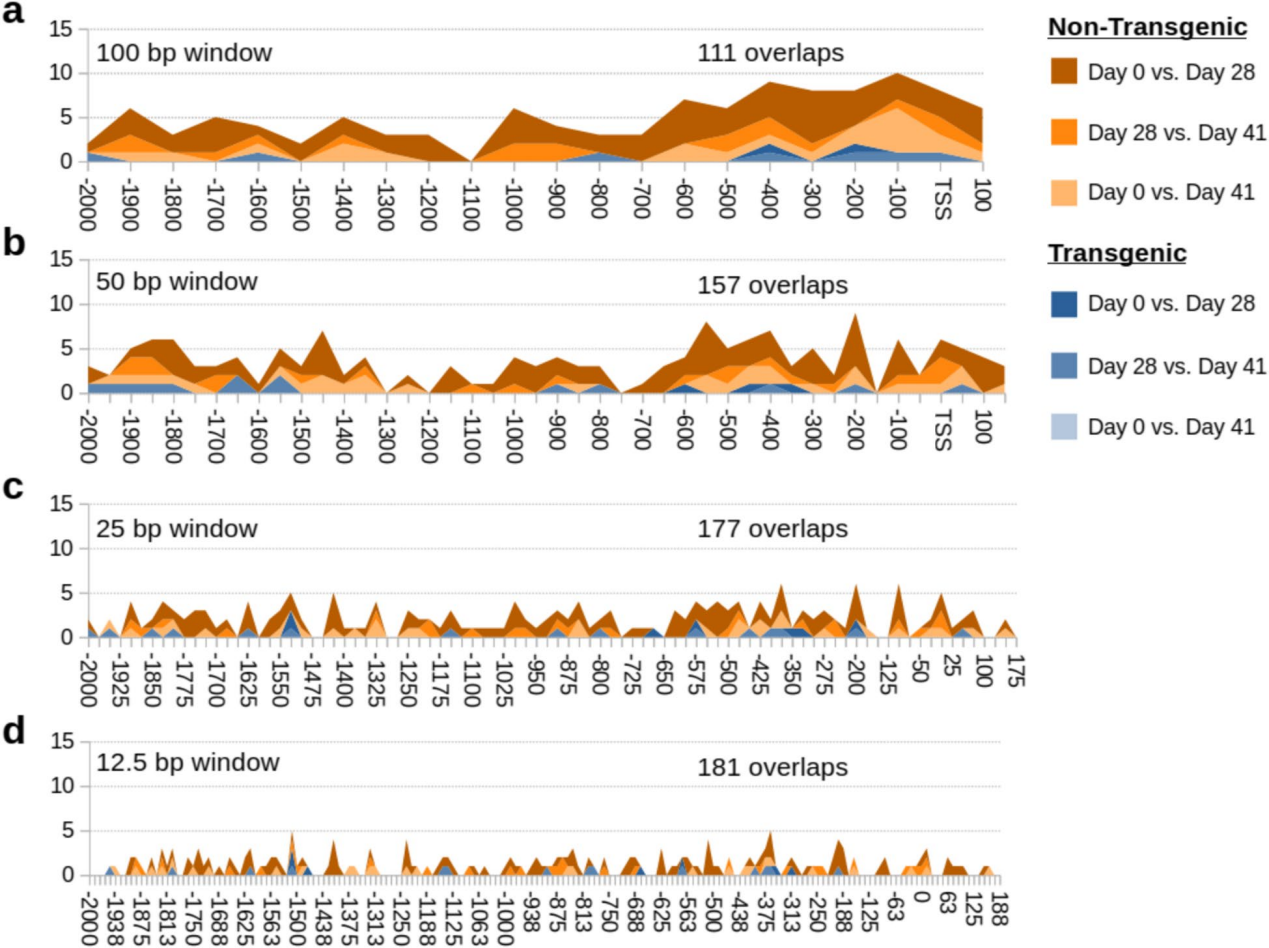
Overlap of differentially expressed genes (DEG) and differentially methylated regions (DMR). Stacked area plot of overlaps of DEGs and DMRs in 100 bp windows (**a**), 50 bp windows (**b**), 25 bp windows (**c**), and 12.5 bp windows (**d**) of the promoter in the same genes. Multiple DMRs from the same gene can overlap with a single DEG

Overlap of significant DEGs and DMRs was uncommon in the promoter as a whole (Table 4) or in windows of the promoter (Fig. 6a-d). There was a ~ 3–4% overlap of DEGs with DMR windows (e.g. for 100 bp windows: 111 overlaps/3106 DMRs = 3.6%). As might be expected, based on the distribution of 100 bp DMRs (Fig. 5), there was an enrichment of overlaps from −600 to + 100 bp of the TSS (Fig. 6a).

**Table 4:** Counts of overlapping DEGs and DMRs in the entire promoter region

Comparison	Promoter
Transgenic Day 0 vs. Day 28	1
Transgenic Day 0 vs. Day 41	0
Transgenic Day 28 vs. Day 41	0
Non-transgenic Day 0 vs. Day 28	7
Non-transgenic Day 0 vs. Day 41	0
Non-transgenic Day 28 vs. Day 41	1
Transgenic vs. non-transgenic Day 0	0
Transgenic vs. non-transgenic Day 28	8
Transgenic vs. non-transgenic Day 41	0

Day 0 = fed, Day28 = feed-deprived, Day 41 = re-fed

## Discussion

Our goals were to determine, 1) if DNA methylation in the promoter was correlated with gene transcription, and 2) if changes in methylation among treatments correlated with changes in gene transcription. We chose to study coho salmon as there were few whole-genome methylation studies in salmonids examining these questions, and because a transgenic model was available that can induce strong physiological effects on phenotype and gene expression (e.g., (Christensen et al. 2021), see (Devlin et al. 2015)). Having both models allowed us to examine if patterns would be expected to be consistent across studies when the physiology of a salmon is altered.

### RNA-Seq Analysis

We observed a strong gene transcription response in liver tissue from feed-deprivation with the percentage of the total number of genes influenced ranging from 3.25% in transgenic salmon to 8.39% in non-transgenic salmon. There was a weaker response after re-feeding, 0.08% in transgenic salmon and 2.85% in non-transgenic salmon. It was previously observed that fully satiated growth hormone (GH) transgenic salmon have decreased stores of hepatic glycogen compared to non-transgenic salmon (Thompson et al. 2023). Salmon from that study were from the same genetic background and had similar body weights as the current study (~ 75 g vs. ~ 100 g). The reduced stores of glycogen might be partially responsible for the difference in gene transcription, or the reduced glycogen storage may point to other factors that influence both glycogen storage and the response in transcription to feed-deprivation. We also note that the age between experimental groups might account in part for these findings (and the minor difference in length observed between groups during the experiment).

A large portion of the differentially expressed genes (DEGs) were shared between non-transgenic and transgenic salmon after feed-restriction and re-feeding, but there were more DEGs among non-transgenic salmon comparisons. The large proportion of shared DEGs is consistent with a previous study comparing stream (with no supplemental feed) and tank environments of coho salmon from the same genetic background (although those salmon were much smaller, ~ 5 g vs. ~ 100 g in the present study) (Christensen et al. 2021). In that study, around 40% of the liver tissue DEGs were shared between environments. Also from that study, the number of DEGs was comparable in non-transgenic and transgenic salmon between stream and tank environments. This suggests a similar, but still unique gene transcription response to feed-restriction between the non-transgenic and transgenic salmon, with non-transgenic salmon having a response involving more gene transcription changes during feed-restriction.

Genes related to the cell cycle are down-regulated after feed-deprivation. The trend of down-regulation is reversed after re-feeding for both groups of salmon. This is a similar response for liver tissue to feed-restriction and re-feeding to that observed in bovines (Keogh et al. 2016). Inhibiting the cell cycle may reduce the need for energy at the expense of growth during feed-restriction. Further salmonid studies will need to be performed to understand if this a common phenomenon.

Major differences between non-transgenic and transgenic salmon during feed-deprivation include changes in genes related to immune response, apoptosis, and sulfur compound metabolic processes. It has previously been reported that regulation of genes related to the immune system are altered in response to feed-deprivation in salmonids (Salem et al. 2007). In non-transgenic Atlantic salmon (*Salmo salar*), roughly half the size of the salmon from the current study (~ 54 g vs. ~ 100 g), there were changes in liver transcription of individual immune-related genes, but not GO term enrichment after starvation (Martin et al. 2010). In the same study, there was up-regulation of genes with functions related to sulfur metabolic process (the opposite direction of regulation from the current study). Neither observation is consistent with the non-transgenic GO term enrichment categories from the present study, suggesting that size, environmental, or species factors might influence the GO term enrichment for these categories more than feed-deprivation.

### Whole Genome Bisulfite Sequencing (WGBS) Analysis

The largest number of differentially methylated CpGs in non-transgenic salmon was identified between the fed time-point and the other two times. In contrast, in transgenic salmon, the largest number of differences in methylation of CpGs was observed between feed-deprivation and re-feeding. Non-transgenic salmon had more overall changes in methylation (~ 2x), with more hyper-methylated markers relative to hypo-methylated markers after feed-deprivation (~ 1.5 × more) and more hypo-methylated markers after re-feeding (~ 1.6 × more). These observations are consistent with the greater number of DEGs identified in non-transgenic salmon.

For all time-points and for both genotypes of coho salmon, we measured methylation levels around the promoter regions of genes. The region upstream of the transcription start site (greater than 2000 bp before the transcription start site—TSS) is highly methylated, however, there is a steep decline in methylation of CpGs closer to the TSS, with an inflection around 200 bp downstream of the start site. In this range, which we refer to as the promoter region in this study (the actual promoter regions of these genes are not known in coho salmon), we noted an association between high methylation and lower gene transcription.

In a meta-analysis of human cancer tissues, the strongest negative correlation between gene transcription and methylation was identified in this region as well (Spainhour et al. 2019). This observation has also been previously described in other studies and species (Brenet et al. 2011; Christensen et al. 2021). We note a finer distinction of this relationship in that only some genes appear to be influenced by promoter region methylation – mostly genes with low or high-levels of methylation. Only genes with high-levels of methylation were negatively associated with gene transcription for all times and genotypes. The association of promoter region methylation and gene transcription was observed before treatment and was consistent after treatments, suggesting that this relationship was the default behaviour within this tissue rather than in response to any environment or genotype effect. From this evidence, it appears that there is a relationship between stable promoter region methylation and gene transcription globally. However, we needed to examine other evidence to understand if changes in methylation impact gene transcription. For example, the number of differentially methylated CpGs was highest after re-feeding in transgenic salmon (~ 43% hypo-methylated and 57% hyper-methylated CpGs), which does not match levels of gene transcription with ~ 19% up-regulated (162) and ~ 81% down-regulated DEGs (700). This suggests that changes in methylation of individual CpGs are independent of changes in gene-transcription as a whole. This could mean that individual CpGs were too fine of a scale to identify associations with gene transcription, that only a subset of genes are impacted by individual CpGs, or that individual CpGs may not influence gene transcription.

### Overlap of DEGs and DMRs

We investigated different regions of the promoter for evidence of overlap between changes in both methylation and gene transcription. We identified limited evidence for these links. For example, in the comparison between fed and feed-deprived non-transgenic salmon, we observed 7 out of 2337 DEGs with significant changes in whole promoter methylation, which is around 0.3% of the DEGs (28% of the DMRs). A similar observation was noted in a study using the same coho salmon model when promoter methylation changes minimally overlapped with changes in genes transcription (Christensen et al. 2021). Changes in gene transcription in response to feed-deprivation were also not correlated with gene methylation in the large yellow croaker (*Larimichthys crocea*) (Zhang et al. 2019) and rainbow trout (Freij et al. 2024). The authors of the large yellow croaker study suggested that methylation might be involved in post-transcriptional modifications rather than gene regulation.

In a meta-analysis of methylation and gene transcription data from human cancer tissues, researchers did not find a general association between methylation and gene transcription (Moarii et al. 2015). Instead, they observed that gene expression of certain transcription factors was linked with promoter methylation making the gene regulatory function of promoter methylation limited to only a subset of genes. While the links between changes in methylation and gene transcription are important to identify and give us further insight into the complex machinery of gene transcription, we interpret that these results point to other mechanisms of gene transcription regulation for the vast majority of changes in genes transcription observed in this study. This could include methylation at regions outside the promoter, which was not examined.

A possible technical explanation for the lack of overlap between DMRs and DEGs is that the criteria and statistical tests were too stringent. This could happen if the variation created by pooling samples caused too much noise to identify all the DMRs and DEGs. For this reason, we also tested if there was a relationship between changes in methylation and transcription using linear regression and for subsets of regions of the promoter. These analyses should be less sensitive to this type of variation.

Most significant inverse associations between changes in methylation and transcription were identified within a few hundred base pairs of the TSS. Given that the average CpG, is around 70% methylated 1000 bp upstream of the TSS in this study, this region is the most likely location to observe an increase of methylation because all the other CpGs outside this region are already mostly methylated. While there were many more significant windows in the non-transgenic salmon comparisons, both salmon genotypes had similar patterns of significant windows. For example, the comparison between the fed (Day 0) and re-fed (Day 41) treatments had the most significant associations in both salmon strains and the comparison of the fed vs. feed-deprived (Day 28) had the least. This supports that these results are robust and that different treatments could have different numbers of genes regulated by DNA methylation. This is further supported by analyses of overlapping DEGs and DMRs in subset windows (the size of the window did not appear to greatly impact the analysis). The vast majority of changes in gene transcription did not appear to be influenced by promoter methylation for any of the methods of analysis used. Also, we note that Freij et al. (2024), observed similar results with less stringent analyses.

## Conclusions

We conclude that promoter methylation in coho salmon was consistently negatively associated with gene transcription before and after treatments but that changes to methylation that accompanied different treatments did not generally correlate with changes in gene transcription. We interpret these results as evidence that methylation has an important gene regulatory function globally (e.g., long-term gene silencing), but that responses to environmental changes (e.g., feed deprivation) and physiological states (e.g., GH transgenesis) in gene transcription are more likely to be mediated by other gene regulatory mechanisms. This suggests that for the majority of changes in methylation we observed, we do not yet fully understand the consequences of those changes for the cell or organism.

## Materials and Methods

To prevent the escape of genetically modified salmon into the wild, all experiments and animal husbandry took place at Fisheries and Oceans Canada West Vancouver facility, which is designed with special containment measures. Experiments were conducted according to the Canadian Council on Animal Care guidelines with the approval of Fisheries and Oceans Canada’s Pacific Regional Animal Care Committee (Animal Use Permit PRACC13-012).

Standard animal husbandry practices for salmon were used for the duration of the experiments with salmon reared at a density of approximately 2.2 kg/m^3^ in 3700L tanks supplied with aerated well water maintained at 10.5 ± 1 °C and a simulated natural photoperiod. When euthanasia was required for sampling, salmon were rapidly euthanized in a bath of tricaine methanesulfonate at a lethal concentration (200 mg/L; Syndel Laboratories Ltd., buffered in 400 mg/L sodium bicarbonate) after an initial sedation using Aquacalm (1 mg/L; Syndel Laboratories Ltd.).

### Experimental Design

The goal of this study was to understand how methylation influences gene transcription, and to understand how the association differs by treatment, group, and how changes in methylation influence changes in gene transcription as a result of different treatments. To accomplish this, we investigated methylation and gene transcription in different lines of salmon after multiple treatments, and we evaluated the relationship between methylation and gene transcription for these groups and treatments. We also examined the changes in methylation and gene transcription between treatments. The changes in methylation between treatments were evaluated to better understand the role of methylation in gene regulation as opposed to the stable down-regulation of gene transcription that might be considered epigenetics. The impact of methylation on gene transcription was investigated in two groups of salmon (non-transgenic and growth hormone (GH) transgenic) to better understand if the results were repeatable in divergent samples.

All coho salmon were initially fed to satiation twice daily with commercial salmonid diet (Skretting Canada Ltd.). At six months of age, transgenic coho salmon (strain M77 – possessing a growth hormone transgene under the control of a constitutive promoter (Devlin et al. 2004)) were the same body size as 19-month non-transgenic coho salmon from the same genetic background (Chehalis River strain, BC Canada). Once the transgenic salmon (103.1 ± 10.72 g) were size-matched with the non-transgenic salmon (102.18 ± 13.85 g), the feed deprivation phase began (day 0). There were about 80 parr (a sub-adult phase in salmon) per genotype reared in separate 3700 L tanks. Feed-deprivation lasted for 28 days. After 28 days, salmon were fed a satiating ration in a manner that they were fed before deprivation (see above).

Experimental groups of salmon were initially size-matched to reduce the influence of developmental stage on gene expression that accompanies differences in body size and developmental stages (White et al. 2013). However, variation in gene expression between the two groups could be a result of differences in age or as a result of the transgene. Our goal is to understand how methylation influences gene transcription rather than comparing non-transgenic and transgenic salmon developmental stages (which has previously been studied). Variation among groups should increase the scope of our understanding of methylation mediated gene transcription.

Weight and length measurements were taken before feed-deprivation (day 0), 1 week after feed deprivation, 4 weeks after feed deprivation (day 28), and 13 days after re-feeding (day 41). Salmon were anaesthetized for measurements in 70 mg/L tricaine methanesulfonate buffered with 140 mg of sodium bicaronate. Nine to 11 randomly selected salmon from each genotype were euthanized at these time-points to sample tissues for RNA-Seq analysis. Length and weight data of the randomly sampled salmon can be found in Figure S1 and Table S1. No significant differences in weight were detected among sampled groups (p = 0.34; 2-way ANOVA). A small genotype effect was detected for length (p = 0.038; 2-way ANOVA). This difference could be a result of developmental factors from age at sampling rather than the influence of the transgene, but the variability should make our findings related to the influence of methylation on gene expression more broad and robust. Liver tissues were collected and fixed in RNAlater using the manufacturer’s protocol (ThermoFisher Scientific). These samples were collected for RNA-seq and whole-genome bisulfite sequencing (described below). The focus of the study was on long-term feed-deprivation and thus samples at one week food deprivation (day 7) were not analyzed further.

### RNA-seq

Two replicates of pools of two individuals each were randomly selected from both transgenic and non-transgenic groups for RNA-seq and bisulfite sequencing (see below) for each treatment group (days 1, 28, and 41; Table S2). Sampled salmon did not differ in weight or length (p > 0.102; 2-way ANOVA). We observed a large deviation in the size of salmon that were pooled, with some larger and smaller samples being paired (Table S2). While this added unintended variability, clustering in MDS plots were mainly as expected using RNA-seq and WGBS data (see Results).

RNA was extracted using an RNeasy Mini Kit (Qiagen) from whole liver tissue. Stranded mRNA libraries were produced at the Centre d'expertise et de services Génome Québec. RNA quality (260/280 absorbance approximately 2.0, RIN > 8) was verified on a NanoDrop ND-1000 Spectrophotometer (NanoDrop Technologies) and a 2100 Bioanalyzer (Agilent Technologies). A NEBNext Poly(A) Magnetic Isolation Module (New England Biolabs—NEB) was used for mRNA enrichment from 250 ng of total RNA and cDNA synthesis was performed using the NEBNext RNA First Strand Synthesis and NEBNext Ultra Directional RNA Second Strand Synthesis Modules (NEB). A NEBNext Ultra II DNA Library Prep Kit for Illumina (NEB) was used for the remaining library steps with adapters and primers from NEB. Library concentrations were quantified using the Kapa Illumina GA with Revised Primers-SYBR Fast Universal kit (Kapa Biosystems). The average fragment size was determined with a LabChip GXII instrument (PerkinElmer). The libraries were sequenced on an Illumina NovaSeq 6000 S4 (PE100).

### Whole Genome Bisulfite Sequencing (WGBS)

From the same liver samples used for RNA preparation, we extracted DNA using the ThermoFisher Scientific Genomic DNA Preparation from RNAlater Preserved Tissue protocol. The genomic DNA was then sent to the Centre d'expertise et de services Génome Québec for WGBS library preparation. DNA was quantified with a Quant-iT PicoGreen dsDNA Assay Kit (Life Technologies). Whole genome shotgun sequencing libraries were generated by using a NEBNext Ultra II DNA Library Prep Kit for Illumina (NEB) with 250 ng of DNA and Adapters from NEB. Libraries were then size selected using sparQ PureMag Beads (Quantabio). The EZ DNA Methylation-Lightning Kit (Zymo Research) was used for bisulfite conversion. Library concentration and size were determined using the same methods as for the RNA-seq libraries. The libraries were sequenced on an Illumina NovaSeq 6000 S4 (PE150). The bisulfite conversion ratio was estimated using telomere sequences with BCREval (Zhou et al. 2020).

### RNA-seq Analysis

Paired-end reads were mapped to the coho salmon reference genome assembly (NCBI accession: GCF_002021735.2, (Rondeau et al. 2022)) after trimming. Trimmomatic (Bolger et al. 2014) was used to remove adapter sequences, trim the 3’ end of a read for a minimum quality of Q30, and for filtering processed reads that were less than 32 bp. STAR (Dobin et al. 2013) was then used to align the remaining paired-end reads to the coho salmon assembly using the two-passes method. HTSeq count (Anders et al. 2015) (intersection-nonempty mode) was then used to count transcripts for each annotated gene or feature from the gene transfer format file provided by the NCBI. We used the RNA-SeQC tool (DeLuca et al. 2012) to determine the quality of the mapping. All prior RNA-Seq analysis steps were implemented using GenPipes (Bourgey et al. 2019).

With the EdgeR (Robinson et al. 2010) package in R (R Core Team 2022), we used the following commands to process the transcript data before pairwise comparisons were made: filterByExpr, calcNormFactors, and estimateDisp (with the robust flag). Differentially expressed genes were identified using the glmFit method in EdgeR with the robust flag (false discovery rate < 0.05). Pairwise comparisons were produced among relevant treatments and groups. All reported transcript values were calculated using the EdgeR function cpm with the log flag (log2) unless otherwise noted. The EdgeR software was chosen for its suitability for our sampling strategy, and it was able to process gene transcription and methylation data.

Enriched GO categories were identified using gProfiler2 (Kolberg et al. 2020) using the gost function (on the web-server, https://biit.cs.ut.ee/gprofiler/gost). This uses a hypergeometric test and a correction for multiple testing (Kolberg et al. 2020). The following parameters were used for the analysis: organism was set to coho salmon (“okisutch”) and only significant results were returned. Only the enriched GO biological process categories were retained (File S1). Enriched GO categories were summarized with REVIGO (Supek et al. 2011) (parameters: small list, species set to Danio rerio, http://revigo.irb.hr/).

### WGBS Analysis

Reads were trimmed using the same method as in the RNA-seq analysis section above. Trimmed reads were aligned to the coho salmon genome assembly (NCBI: GCF_002021735.2) with Bismark (Krueger and Andrews 2011). PCR duplicates were removed using Picard (2020) and methylation counts (from both strands) for all CpG sites were calculated using the Bismark function Bismark_methylation_extractor. The methylation counts were imported into the R package MethylKit (Akalin et al. 2012). Using the MethylKit package, we removed CpG sites with less than 10 total read coverage for each pool of samples (to reduce the influence of variation in sequence coverage). CpG sites were retained if they had adequate coverage for all samples (13.8 million sites were retained). We then normalized coverage across individual pools using the normalizeCoverage function in MethylKit. Pairwise comparisons between time-points and genotypes were calculated using the MethylKit package using a logistic regression with overdispersion correction for all CpG sites. CpG sites positions were annotated and only CpGs with a genomic annotation were reported for all analyses.

We also analyzed the methylation data based on promoter region (defined in this study for convenience as 2000 bp upstream and 200 bp downstream of transcriptional start site—core regulatory elements are commonly found within a few hundred basepairs of transcriptional start sites in humans, e.g., (FitzGerald et al. 2004; Cooper et al. 2006; Benner et al. 2013)). This included the entire promoter and windows of the promoter (100 bp, 50 bp, 25 bp, and 12.5 bp). For these analyses, we summed the counts of methylation marks for each window after normalization in EdgeR. The regions were then used for differential methylation region analysis in EdgeR (see File S2 for more details). Our threshold for significance was a q-value of less than 0.01 and a 25% difference in methylation values for differential methylation analyses for the entire promoter, or a *p*-*value* < 0.05 for all other windows (to investigate the effect of using a less stringent threshold). See File S2 for example code. Associations between gene transcription and promoter methylation were evaluated by overlaps in DEGs and DMRs (i.e., the same gene was differentially methylated and expressed) or by linear regression (*p* ≤ 0.05 threshold). See File S2 for example code.

## Supplementary Information

Below is the link to the electronic supplementary material.

Supplementary file1 Scripts used in manuscript -- in compressed format (TAR.GZ 9 KB)

Supplementary file2 Weight and length of sampled salmon during periods of feeding (Day 0), feed-deprivation (Days 7 and 28) and re-feeding (Days 28-41) (PDF 70 KB)

Supplementary file3 RNA-seq quality control a) Aligned read counts (single). b) PCA of most differentially expressed genes. Distance corresponds to leading log-fold-change between each pair of samples. c) MDS plot of all genes. Distance corresponds to the leading log-fold-change (PDF 81 KB)

Supplementary file4 WGBS quality control a) MDS plot of the top 10,000 most variable CpGs, b) MDS plot of all CpGs. c) MDS plot of the top 10,000 most variable promoter regions (based on the fraction of methylation). d) MDS plot of all promoter regions (PDF 119 KB)

Supplementary file5 Effect on methylation of CpG position relative to the transcription start site of all assayed genes The first panel (PDF) shows the three treatments (fed, feed-restriction, and re-feeding) of non-transgenic salmon. The X-axes show the relative position of CpG loci to the transcription start site. The Y-axes is the percent methylation for each CpG loci averaged for each pool of each treatment. The second panel is the same, but for transgenic salmon (PDF 254 KB)

Supplementary file6 Gene transcription and promoter methylation in liver tissue from transgenic and non-transgenic salmon during different treatments The relationship between promoter (2000 bp upstream of the transcriptional start site and 200 bp downstream) methylation and gene transcription (log2 of the counts per million mapped reads – log2 CPM) for different treatments. Only genes with values for both were plotted. The ggplot2 (Wickham 2016) command geom_smooth was used to model the data (blue line). On the first panel, non-transgenic salmon are shown for the three treatments with methylation measured from the promoter region. On the second panel, transgenic salmon are shown for the three treatments with methylation measured for the promoter region (PDF 1192 KB)

Supplementary file7 Significant association of the change in promoter region methylation and gene transcription. Scatter plot of the change in methylation (X-axis, window -113 to -125 bp) and the log2 fold-change in gene transcription for each gene in non-transgenic salmon (fed vs. feed-deprived) (PDF 77 KB)

Supplementary file8 Significant DEGs (comparisons in different tabs) and GO terms (XLSX 128 KB)

Supplementary file9 Supplemental tables (DOCX 15 KB)

## Data Availability

All raw sequence data is available under the BioProject ID PRJNA928760 from the NCBI.
