# Supplementary figures and images for: Transcription Dynamics and DNA Methylation Responses to Growth Modification
Source: Mar Biotechnol (NY). 2025 Jun 12;27(3):96. doi: 10.1007/s10126-025-10476-3 (PMC12162792; doi:10.1007/s10126-025-10476-3)

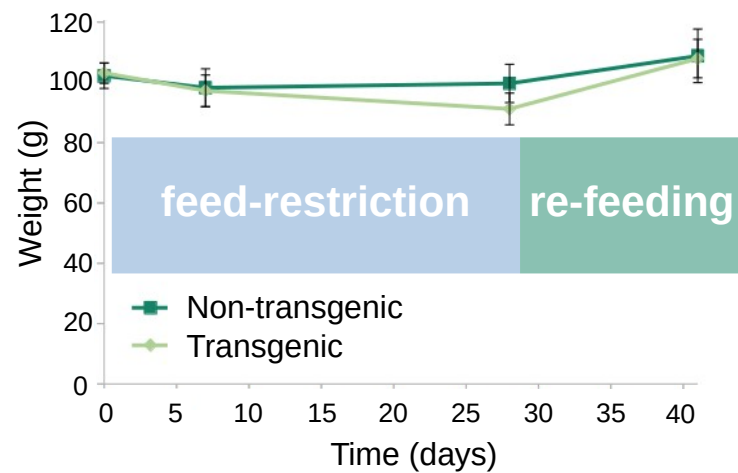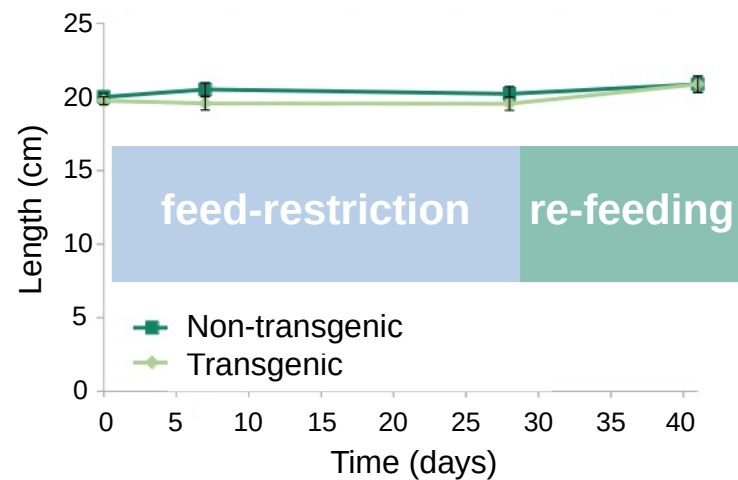

Supplement: Supplementary file 2 — Supplementary file2 Weight and length of sampled salmon during periods of feeding (Day 0), feed-deprivation (Days 7 and 28) and re-feeding (Days 28-41) (PDF 70 KB) [file 10126_2025_10476_MOESM2_ESM.pdf]

**a**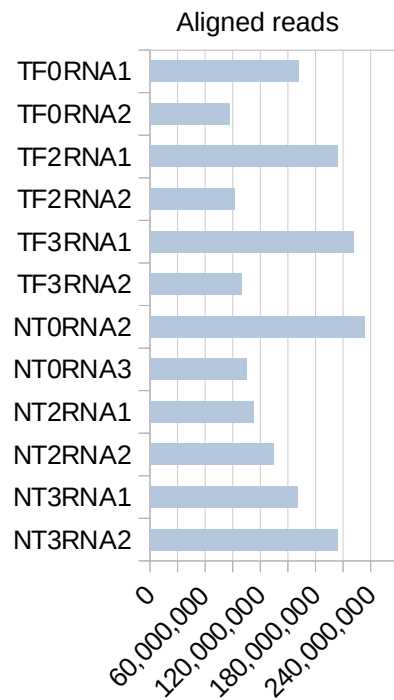**b**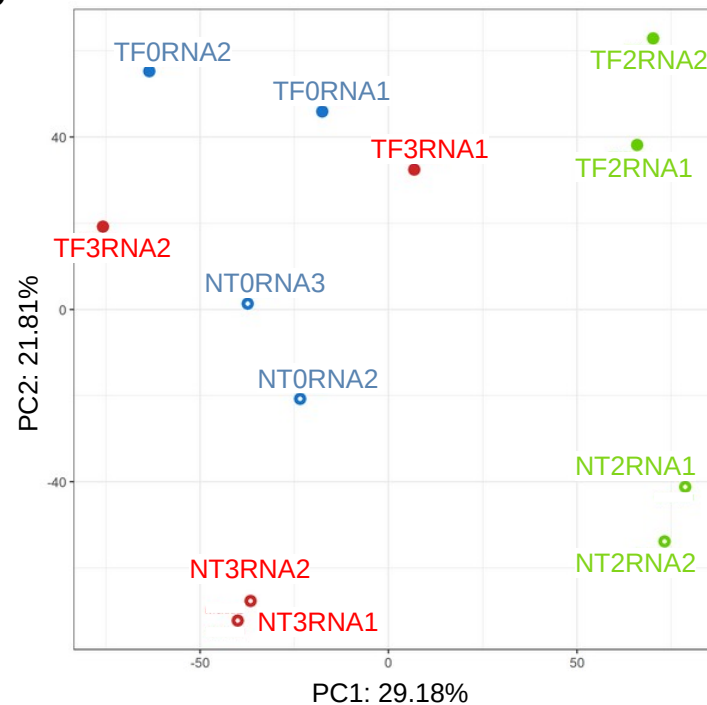**c**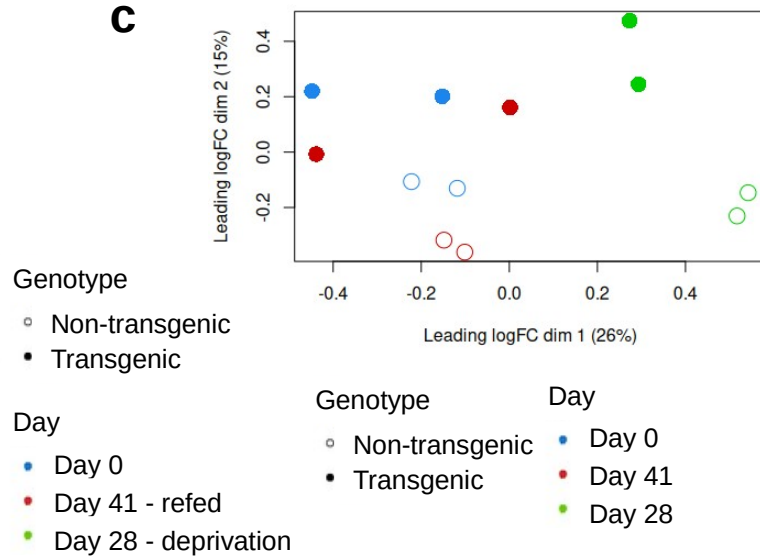

Supplement: Supplementary file 3 — Supplementary file3 RNA-seq quality control a) Aligned read counts (single). b) PCA of most differentially expressed genes. Distance corresponds to leading log-fold-change between each pair of samples. c) MDS plot of all genes. Distance corresponds to the leading log-fold-change (PDF 81 KB) [file 10126_2025_10476_MOESM3_ESM.pdf]

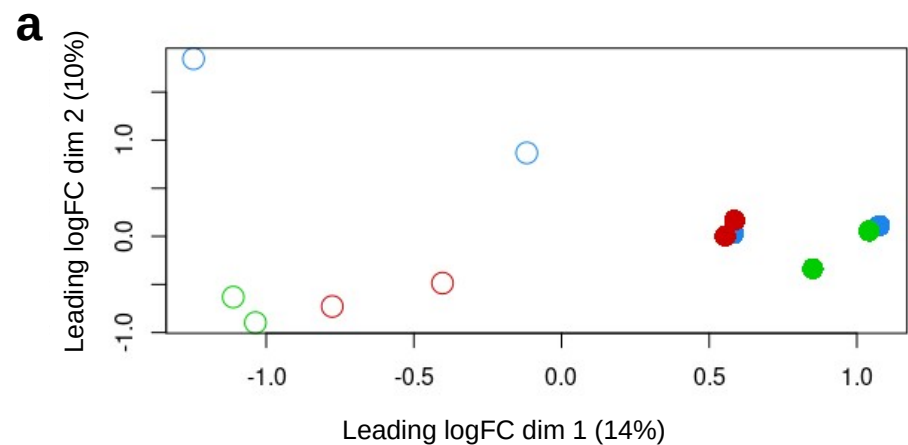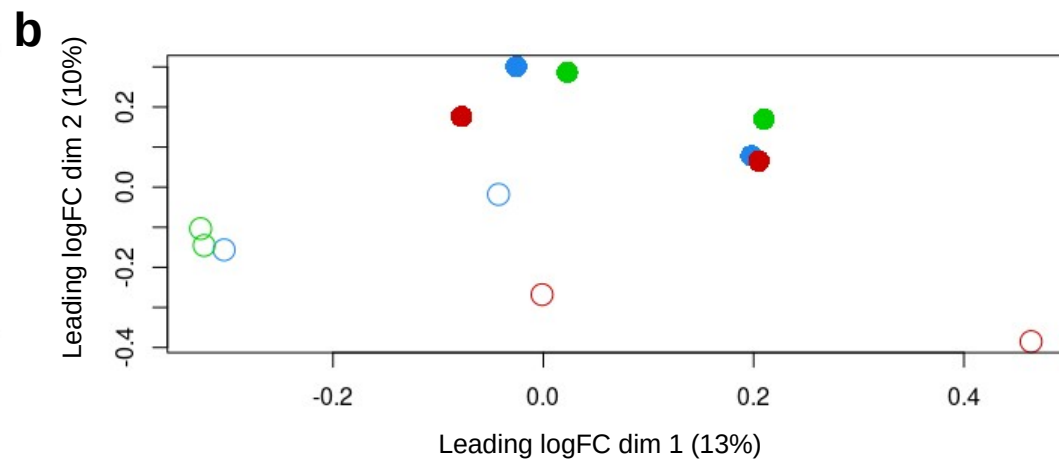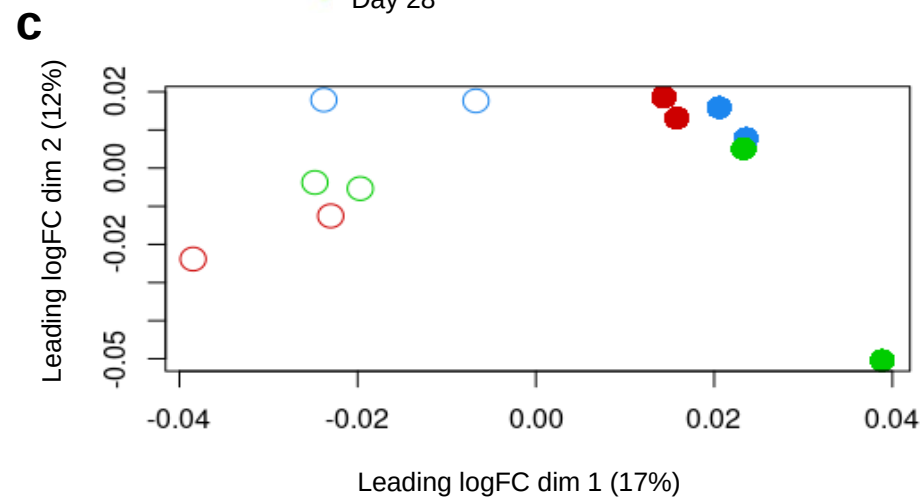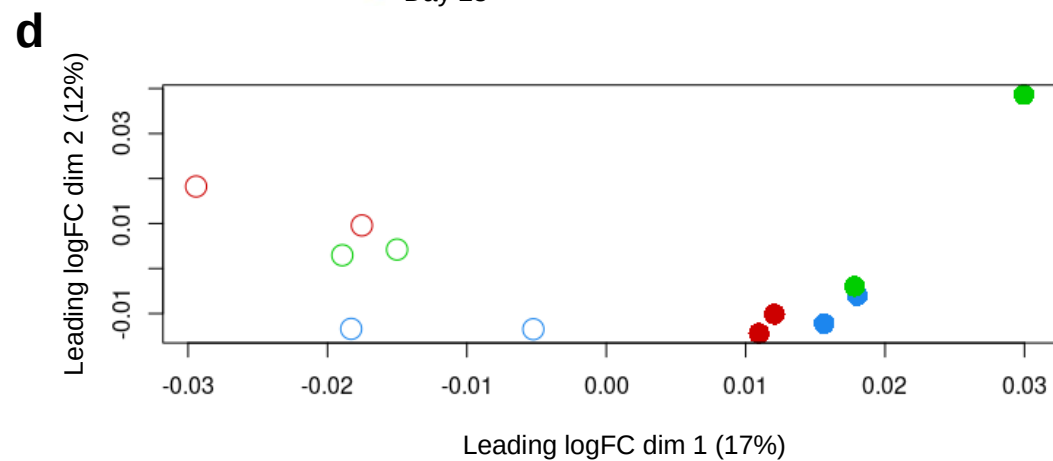

Supplement: Supplementary file 4 — Supplementary file4 WGBS quality control a) MDS plot of the top 10,000 most variable CpGs, b) MDS plot of all CpGs. c) MDS plot of the top 10,000 most variable promoter regions (based on the fraction of methylation). d) MDS plot of all promoter regions (PDF 119 KB) [file 10126_2025_10476_MOESM4_ESM.pdf]

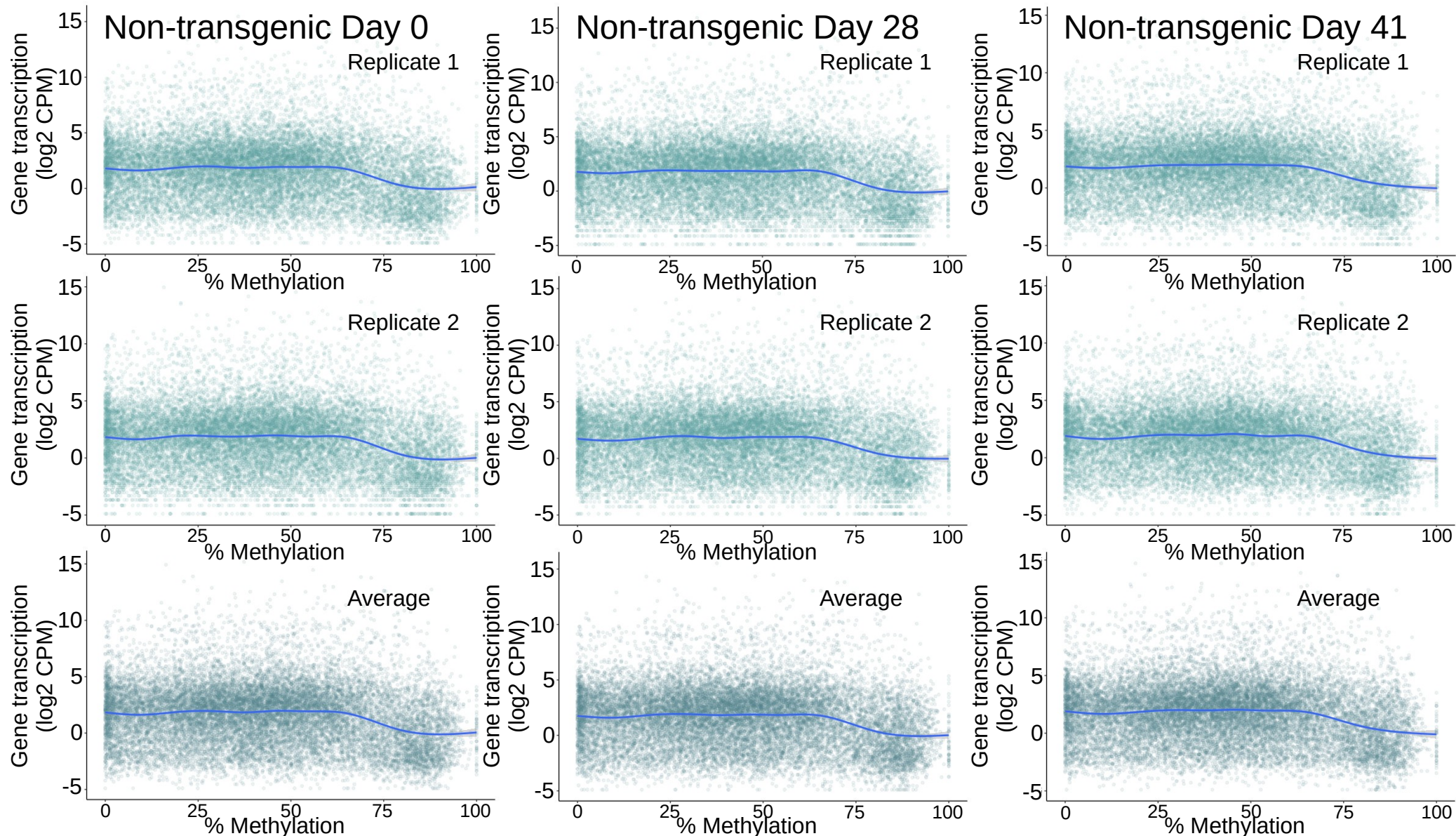

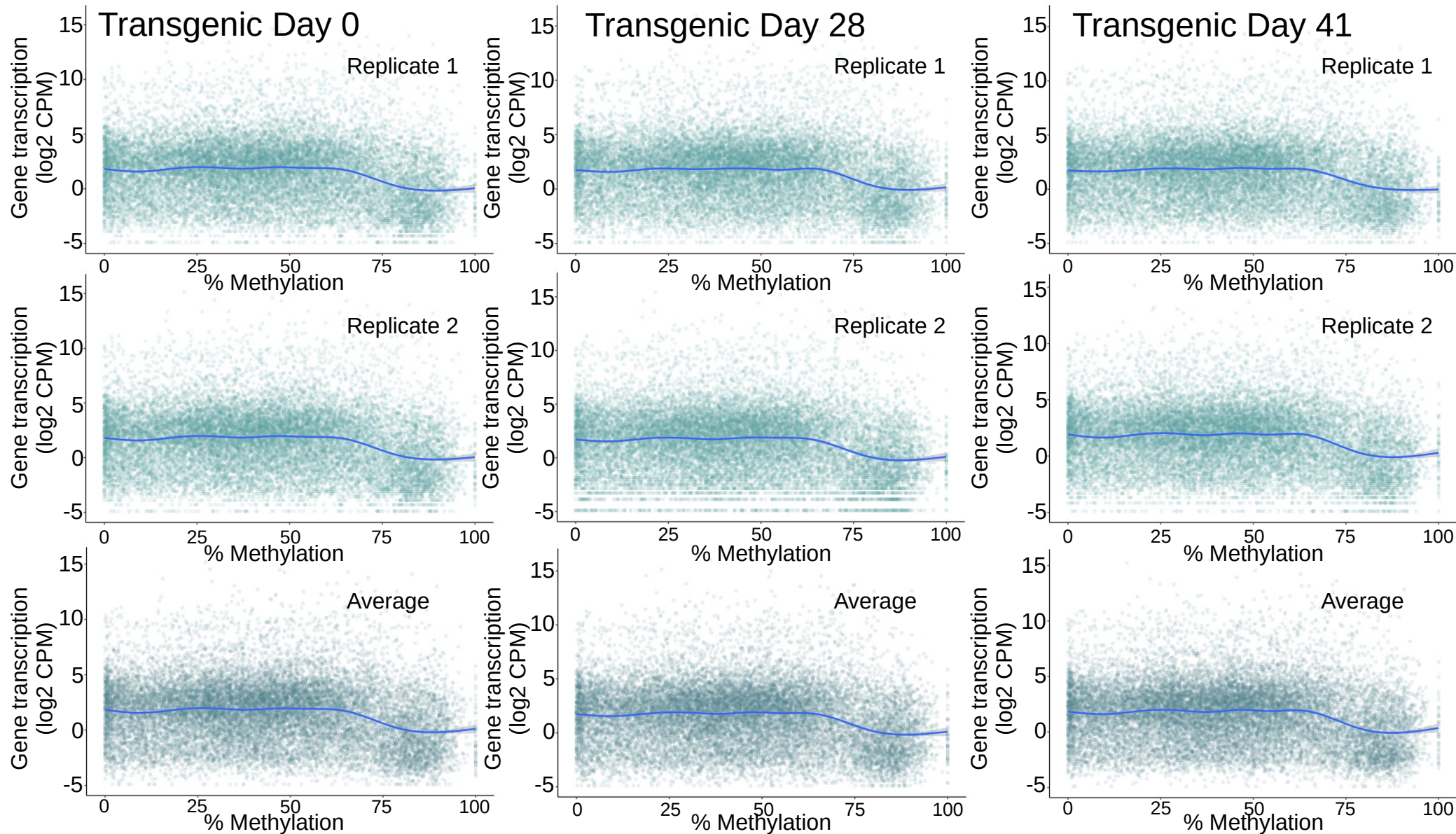

Supplement: Supplementary file 6 — Supplementary file6 Gene transcription and promoter methylation in liver tissue from transgenic and non-transgenic salmon during different treatments The relationship between promoter (2000 bp upstream of the transcriptional start site and 200 bp downstream) methylation and gene transcription (log2 of the counts per million mapped reads – log2 CPM) for different treatments. Only genes with values for both were plotted. The ggplot2 (Wickham 2016) command geom_smooth was used to model the data (blue line). On the first panel, non-transgenic salmon are shown for the three treatments with methylation measured from the promoter region. On the second panel, transgenic salmon are shown for the three treatments with methylation measured for the promoter region (PDF 1192 KB) [file 10126_2025_10476_MOESM6_ESM.pdf]

Window -113 to -125 bp  
Non-transgenic

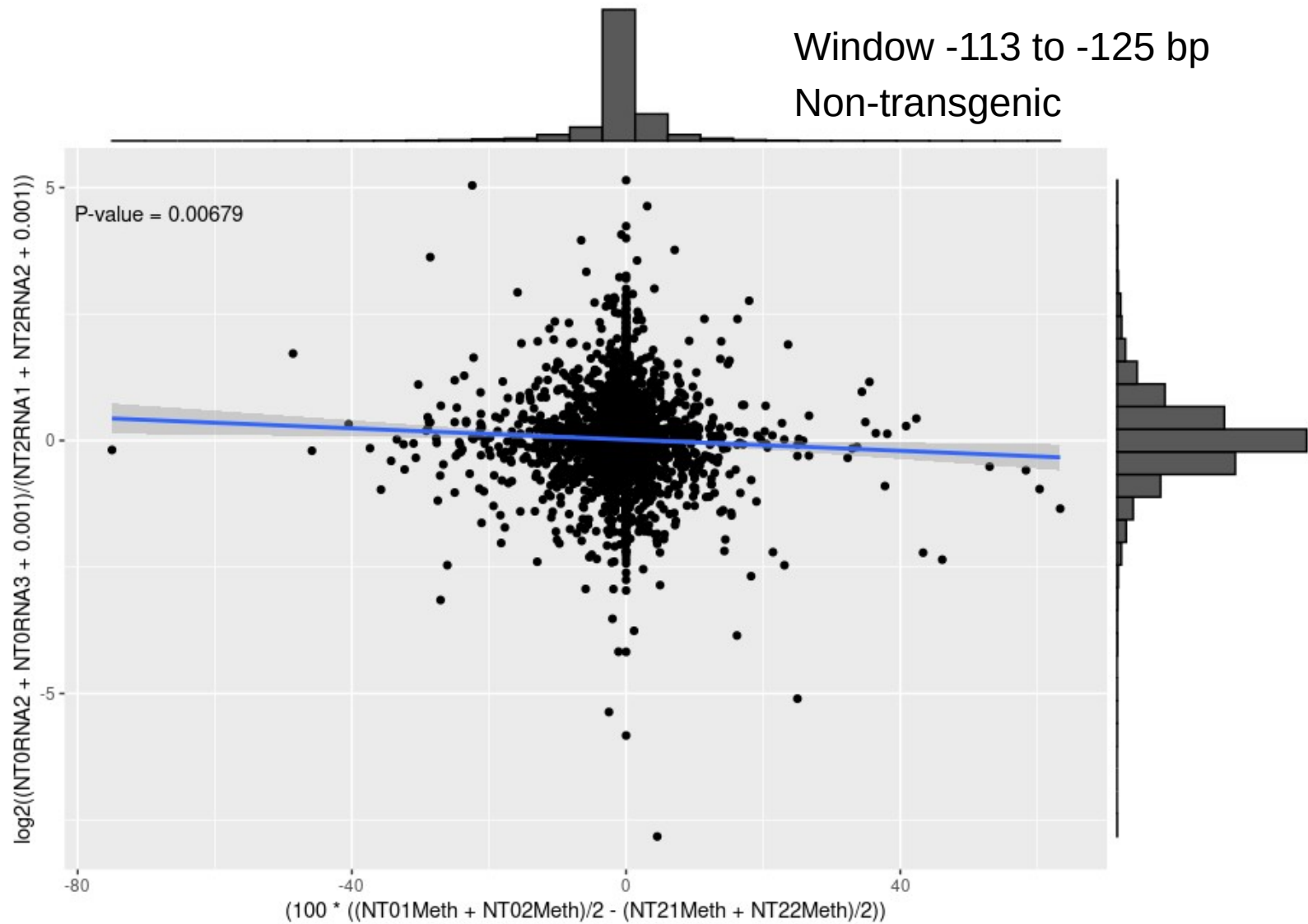

Supplement: Supplementary file 7 — Supplementary file7 Significant association of the change in promoter region methylation and gene transcription. Scatter plot of the change in methylation (X-axis, window -113 to -125 bp) and the log2 fold-change in gene transcription for each gene in non-transgenic salmon (fed vs. feed-deprived) (PDF 77 KB) [file 10126_2025_10476_MOESM7_ESM.pdf]
