# Supplementary material for: Transcription Dynamics and DNA Methylation Responses to Growth Modification
Source: Mar Biotechnol (NY). 2025 Jun 12;27(3):96. doi: 10.1007/s10126-025-10476-3 (PMC12162792; doi:10.1007/s10126-025-10476-3)
Supplement: Supplementary file 5 — Supplementary file5 Effect on methylation of CpG position relative to the transcription start site of all assayed genes The first panel (PDF) shows the three treatments (fed, feed-restriction, and re-feeding) of non-transgenic salmon. The X-axes show the relative position of CpG loci to the transcription start site. The Y-axes is the percent methylation for each CpG loci averaged for each pool of each treatment. The second panel is the same, but for transgenic salmon (PDF 254 KB) [file 10126_2025_10476_MOESM5_ESM.pdf]

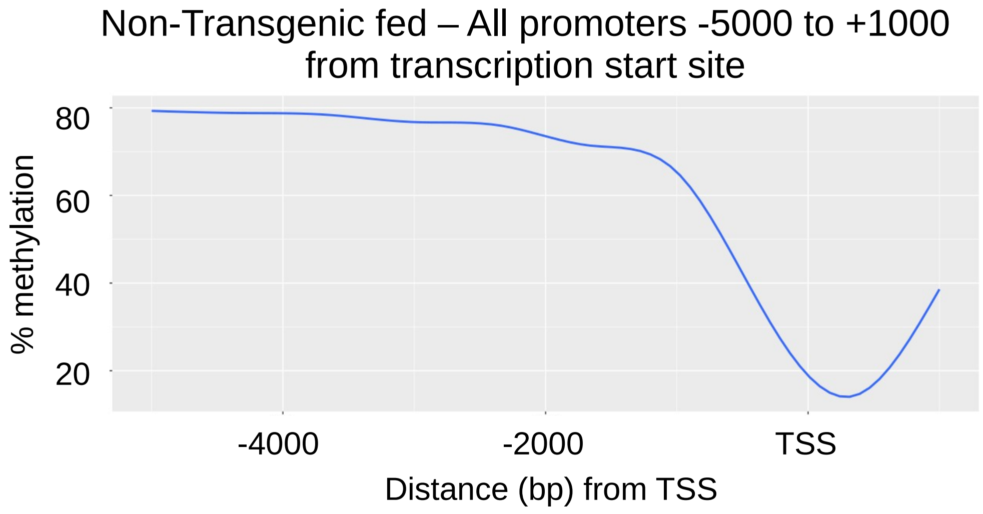
Non-Transgenic fed – All promoters -5000 to +1000 from transcription start site
80
60
40
20
% methylation
-4000
-2000
TSS
Distance (bp) from TSS

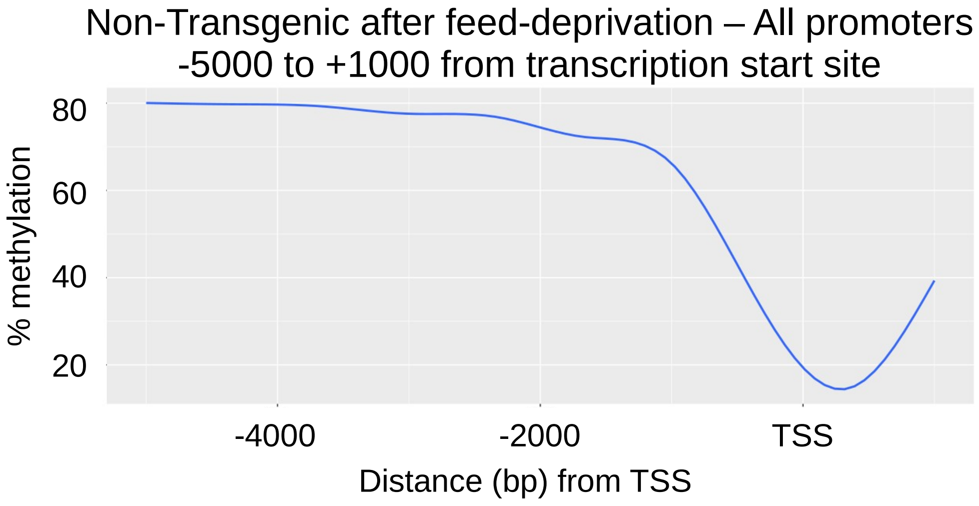
Non-Transgenic after feed-deprivation – All promoters -5000 to +1000 from transcription start site
80
60
40
20
% methylation
-4000
-2000
TSS
Distance (bp) from TSS

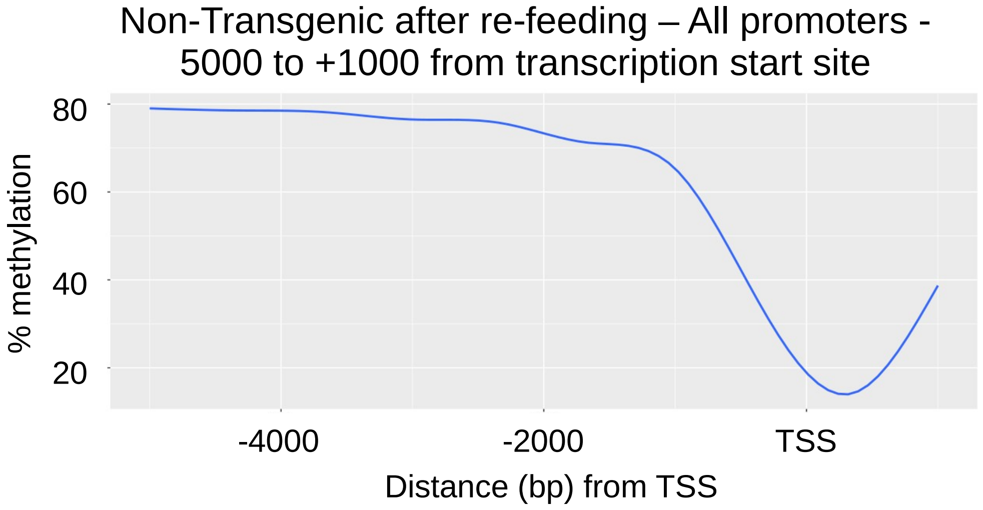
Non-Transgenic after re-feeding – All promoters -5000 to +1000 from transcription start site
80
60
40
20
% methylation
-4000
-2000
TSS
Distance (bp) from TSS

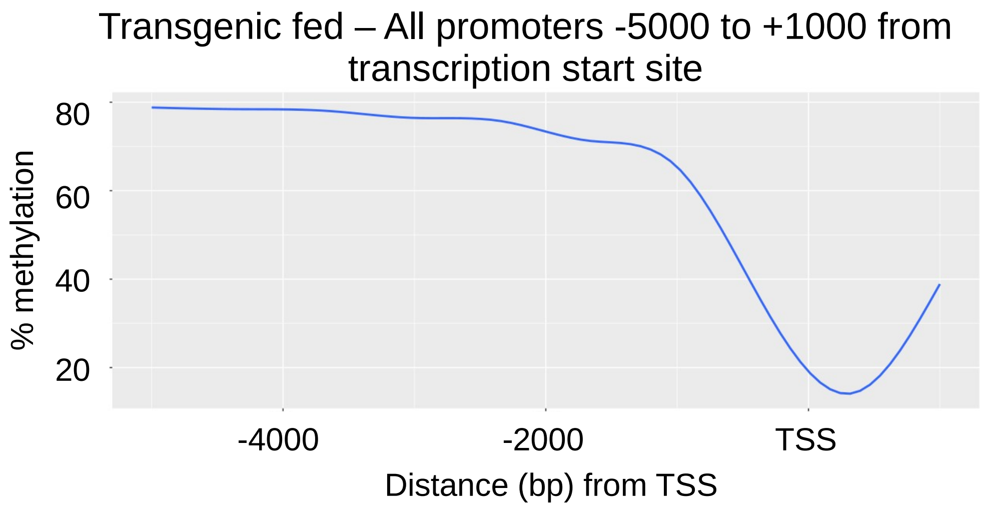
Transgenic fed – All promoters -5000 to +1000 from transcription start site
% methylation
80
60
40
20
-4000
-2000
TSS
Distance (bp) from TSS

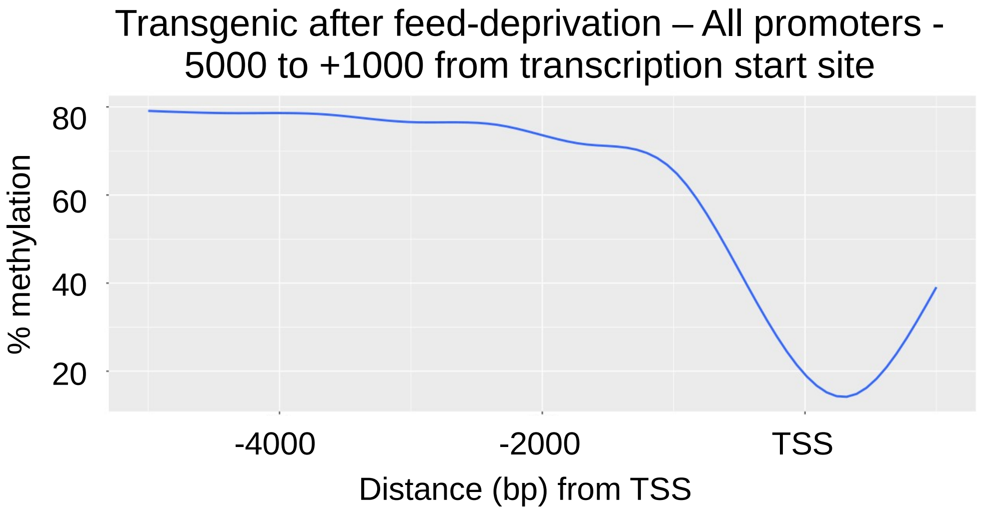
Transgenic after feed-deprivation – All promoters -5000 to +1000 from transcription start site
% methylation
80
60
40
20
-4000
-2000
TSS
Distance (bp) from TSS

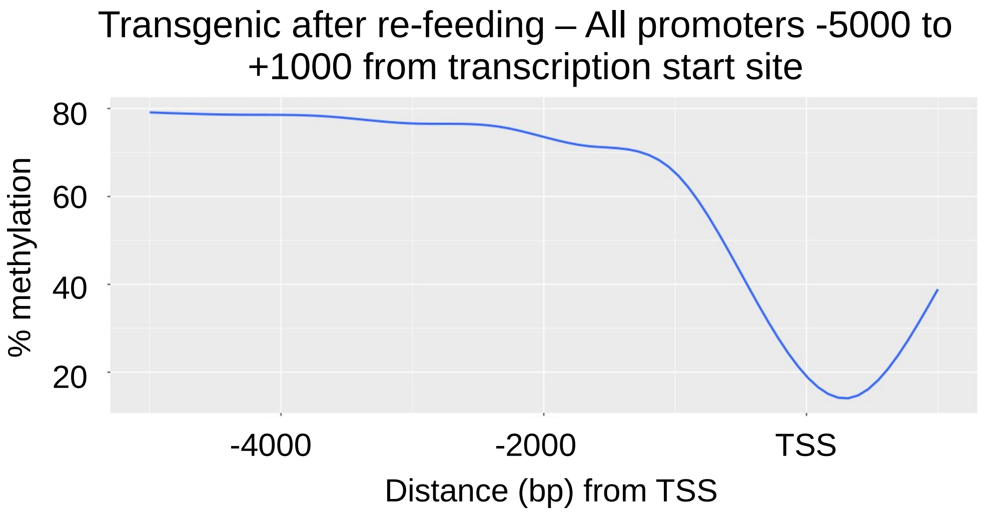
Transgenic after re-feeding – All promoters -5000 to +1000 from transcription start site
% methylation
80
60
40
20
-4000
-2000
TSS
Distance (bp) from TSS
