# Supplementary material for: Transcription Dynamics and DNA Methylation Responses to Growth Modification
Source: Mar Biotechnol (NY). 2025 Jun 12;27(3):96. doi: 10.1007/s10126-025-10476-3 (PMC12162792; doi:10.1007/s10126-025-10476-3)
Supplement: Supplementary file 9 — Supplementary file9 Supplemental tables (DOCX 15 KB) [file 10126_2025_10476_MOESM9_ESM.docx]

Table S1. **Weights (g) of sampled salmon during periods of feeding (Day 0), food deprivation (Days 7 and 28) and refeeding (Day 41).**

| **Weight (g)** | **Genotype** | **Statistic** | **Day 0** | **Day 7** | **Day 28** | **Day 41** |
| --- | --- | --- | --- | --- | --- | --- |
|  | Non-transgenic | average | 102.18 | 98.22 | 99.66 | 108.80 |
|  |  | stdev | 13.85 | 18.81 | 20.08 | 28.09 |
|  |  | count | 11 | 9 | 10 | 10 |
|  | Transgenic | average | 103.10 | 97.20 | 91.20 | 107.90 |
|  |  | stdev | 10.72 | 16.61 | 16.61 | 20.17 |
|  |  | count | 10 | 10 | 10 | 10 |
| **Length (cm)** | Non-transgenic | average | 20.00 | 20.51 | 20.21 | 20.86 |
|  |  | stdev | 0.80 | 1.34 | 1.58 | 1.74 |
|  |  | count | 11 | 9 | 10 | 10 |
|  | Transgenic | average | 19.74 | 19.56 | 19.54 | 20.86 |
|  |  | stdev | 0.85 | 1.43 | 1.43 | 1.74 |
|  |  | count | 10 | 10 | 10 | 10 |
|  |  |  |  |  |  |  |

Table S2. **Genotype, pool number, and body size of salmon sampled on Days 0, 28, and 41.**

| **Day** | **Genotype** | **ID** | **Pool** | **Weight (g)** | **Length (cm)** |
| --- | --- | --- | --- | --- | --- |
| 0 | NT | NT6 | 1 | 78 | 18.5 |
| 0 | NT | NT9 | 1 | 118 | 21 |
| 0 | NT | NT1 | 2 | 102 | 19.9 |
| 0 | NT | NT8 | 2 | 100 | 20.3 |
| 0 | T | TF3 | 3 | 93 | 18.8 |
| 0 | T | TF6 | 3 | 102 | 19.3 |
| 0 | T | TF4 | 4 | 108 | 20.4 |
| 0 | T | TF10 | 4 | 106 | 19.8 |
| 28 | NT | NT3 | 5 | 77 | 18.9 |
| 28 | NT | NT13 | 5 | 106 | 21.5 |
| 28 | NT | NT2 | 6 | 125 | 22.2 |
| 28 | NT | NT9 | 6 | 80 | 19.3 |
| 28 | T | TF2 | 7 | 94 | 20.8 |
| 28 | T | TF3 | 7 | 122 | 22 |
| 28 | T | TF4 | 8 | 69 | 18.2 |
| 28 | T | TF9 | 8 | 113 | 20.6 |
| 41 | NT | NT2 | 9 | 138 | 23 |
| 41 | NT | NT5 | 9 | 109 | 20.8 |
| 41 | NT | NT4 | 10 | 84 | 19.3 |
| 41 | NT | NT10 | 10 | 154 | 23.3 |
| 41 | T | TF2 | 11 | 107 | 20.5 |
| 41 | T | TF4 | 11 | 142 | 22 |
| 41 | T | TF6 | 12 | 101 | 20.2 |
| 41 | T | TF9 | 12 | 128 | 21 |
|  |  |  |  |  |  |

NT = Non-transgenic and T = Transgenic
